# 2877. Influence of vancomycin AUC monitoring on acute kidney injury in a large quasi-experimental study of over 3000 patients

**DOI:** 10.1093/ofid/ofad500.154

**Published:** 2023-11-27

**Authors:** Eric Gregory, Aric Schadler, David Burgess, Donna R Burgess, Sarah Cotner, Jeremy VanHoose, Katie L Wallace

**Affiliations:** The University of Kansas Health System, Kansas City, MO; University of Kentucky Healthcare, Lexington, Kentucky; UK HealthCare, Lexington, KY; UK HealthCare, Lexington, KY; UK HealthCare, Lexington, KY; UK HealthCare, Lexington, KY; University of Kentucky HealthCare, Lexington, KY

## Abstract

**Background:**

Due to the inconsistent correlation of vancomycin trough concentrations with area under the curve (AUC) and increased rates of vancomycin-induced kidney injury (VIKI), an institutional practice change was implemented in 2017 to use 2-level AUC monitoring. The objective of the present study was to evaluate VIKI outcomes in a large quasi-experimental study.

**Methods:**

A 4-year quasi-experimental study was performed at an academic medical center and included patients who received parenteral vancomycin for ≥ 72 hours. The primary outcome was the incidence of VIKI defined using KDIGO criteria. Secondary outcomes included inpatient all-cause mortality, median length of stay (LOS), median intensive care unit LOS, and median vancomycin trough concentration. Multivariable logistic regression analyses were used to identify primary predictors of VIKI and all-cause inpatient mortality while controlling for other significant covariates.

**Results:**

The study included 3207 patients, with 1964 and 1243 in the trough and AUC groups, respectively. Similar baseline demographics (i.e., age, sex, race, weight, BMI, CrCl, Charlson Comorbidity Index) were noted between the groups. Patients in the AUC group were more likely to receive a vancomycin loading dose (32% vs 49%; p< 0.001) and have greater median trough concentrations (11.5 mg/L [8.1-16.0] vs 11.8 mg/L [8.9-16.2]; p=0.008). However, they also had lower median day 3 vancomycin exposures (3500 mg [2250-5000] vs 3250 mg [2500-4500]; p=0.008). Median AUC was 488 (396-607) in the AUC group. No significant differences were noted in the bivariate analysis of VIKI, defined as meeting criteria of any of the 3 KDIGO stages (20.5% vs 19.6%; p=0.112). The multivariable logistic regression suggested AUC monitoring was associated with a 23% decrease in VIKI (OR 0.767; 95% CI 0.621-0.947). All-cause inpatient mortality was significantly decreased in the AUC group (8.1% vs 6.3%; p=0.049).

**Figure:**
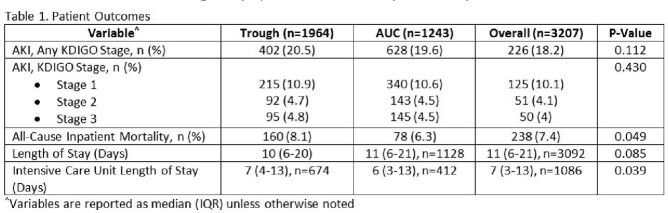

**Conclusion:**

VIKI was significantly reduced when AUC monitoring was employed in a large quasi-experimental study comprising over 3000 patients, one of the largest evaluations to date. This further strengthens the recommendation to transition to AUC monitoring for most patients receiving vancomycin for ≥ 72 hours.

**Disclosures:**

**All Authors**: No reported disclosures

